# *Neuromorpha vorax*: a previously unculturable cosmopolitan protist with an unexpectedly complex life cycle belonging to Glissomonadida Clade-U/Group-TE

**DOI:** 10.1128/mbio.00848-25

**Published:** 2025-05-30

**Authors:** Gabrielle Corso, Lindsay R. Triplett, Daniel J. Gage

**Affiliations:** 1 Department of Molecular and Cell Biology, The University of Connecticut, Storrs, Connecticut, USA; 2 Department of Environmental Science, The Connecticut Agricultural Experiment Station, New Haven, Connecticut, USA; 3 Department of Plant Pathology and Ecology, The Connecticut Agricultural Experiment Station, New Haven, Connecticut, USA; University of Wisconsin-Madison, Madison, Wisconsin, USA

**Keywords:** protists, soil biology, rhizosphere, microbial communities

## Abstract

Soil protists are increasingly recognized as common members of complex communities that associate with plant root systems, though their contributions to these communities and to the plant host remain obscure. Members of Clade-U/Group-TE are cosmopolitan soil protists and often among the most abundant protists associated with plant roots. Here, we describe the isolation, culturing, and characterization of *Neuromorpha vorax*, a member of the previously uncultured Clade-U/Group-TE branch of the order Glissomonadida. *N. vorax* grew readily when provided bacteriophage lysate as a food source. This allowed us to grow large numbers of the organisms from single-cell isolates and provided ideal conditions for following transitions from one morphology to another. *N. vorax*, like most glissomonads, has a small, flagellated gliding form, but it also displays a wide range of other morphologies, including a crawling form, small and large trophozoites with multiple long filopodia, small and large resting cysts, and clusters of large dividing cells, and cannibalistic feeding behaviors. Given the small size of most glissomonads, it may be that other members of this important group, known from environmental surveys but currently uncultured, might also be readily grown on bacteriophage lysates. In addition, given an abundant food source and clear viewing by microscopy, glissomonads and other small protists may be found to have life cycles and behaviors that are more complex than is currently appreciated.

**IMPORTANCE:**

Protists from the Clade-U/Group-TE cluster of glissomonads are widespread and abundant colonizers of plant roots. Despite being known for over 30 years, they have remained uncultured. We show that these protists can be easily cultured using an unusual food source, viral lysates of bacteria. This culturing method allows growth of high numbers of these organisms and reveals that they have an unexpectedly complex lifecycle that includes community feeding and cannibalism. Some other currently unculturable protists can perhaps be grown with these methods, and many of these may also show unexpectedly complex lifecycles. The growth of eukaryotes on virus lysates raises the possibility that viruses in soil may directly contribute to the growth (and not just death) of eukaryotes in soil and root-associated communities.

## INTRODUCTION

The Glissomonadida, an order of protists in the phylum Cercozoa, are generally considered bacterivorous and often among the most abundant protists in terrestrial ecosystems (1–7). They are genetically diverse but morphologically rather uniform. The Glissomonadida consists of roughly a dozen distinct clades. Of these, no member of clades U, Y, Z, and Group-TE has yet to be cultured or characterized beyond sequencing of 18S rRNA genes from environmental gene surveys. Glissomonads are usually small (<10 µm) biflagellate that glide along surfaces using a posterior flagellum and are considered to have only weak amoeboid characteristics (8–10). There are exceptions to this rule of uniformity. *Proleptomonas faecicola* grows as clusters of dividing cells (11); *Saccharomycomorpha* grows osmotrophically as clusters of dividing cells with no gliding form (12); *Orciraptor* and *Viridiraptor* display a wide range of morphologies, including gliding and crawling forms, motile amoeboid forms, several forms capable of cell division, and amoeboid forms that can dissolve algal cell walls and extract the cytoplasm for food (13). These exceptions suggest that glissomonads are more diverse in morphology and behavior than currently thought.

Glissomonads designated as belonging to Group-TE have been repeatedly detected in protist communities from around the world but have resisted efforts to be isolated and cultured. These protists are enriched in rhizosphere samples relative to bulk soil and are often among the most abundant protists in these studies (2, 3, 5–7, 14, 15). As described below, we found that protists designated as members of Group-TE in databases and literature fall into two well-separated clades: a minority belong to Group-TE as designated by Howe et al. (9, 10), but the majority actually belong to Clade-U as designated by Howe et al.

In this paper, we describe the isolation, growth, and characterization of *Neuromorpha vorax*, a member of the previously uncultured Clade-U (a.k.a. Group-TE) protists. *N. vorax* displays a complex life cycle with gliding and crawling forms, several amoeboid feeding forms, several routes of reproduction, and resting cysts. It also displays communal, cannibalistic behavior during feeding.

## RESULTS

### Identification, purification, and culturing of *N. vorax*

A nested PCR approach was used to identify and track Clade-U organisms as complex protist communities from *Zea mays* rhizosphere samples went through repeated cycles of serial dilution and growth. This eventually resulted in an enrichment culture that contained only a Clade-U Glissomonad, and larger protists belonging to *Paratetramitus* were in the culture, as determined by community sequencing of 18S rRNA gene sequences. Repeated attempts to separate Clade-U protists from *Paratetramitus* by dilution and single-cell isolations were unsuccessful probably because Clade-U protists were separated from their food source. Bacteria and bacterial spores purified from the sample did not allow growth of isolated Clade-U protists when added to their culture medium. Given the small size of the Clade-U cells in the enrichment culture (<4 µm body length), we attempted to grow them using bacteriophage as a food source. Bacteriophage lysates made by infecting *Sinorhizobium meliloti* with phage N3 allowed robust growth of the Clade-U protists while unexpectedly eliminating *Paratetramitus* and suppressing visible growth of bacteria. It is unclear why phage N3 at high titer (~10^10^ PFU/mL in this case) suppressed the growth of *Paratetramitus* and bacteria that were not *S. meliloti*. We speculate that the very high multiplicity of infection could have damaged bacteria, or a >100 kD signal in the lysate caused bacteria to enter a non-growing state. We noticed that a high titer (10^11^ PFU/mL) of Actinophage “Dulcie” inhibited the growth of *N. vorax*. Likewise, the high-titer N3 lysate may have damaged *Paratetramitus*, or this protist may have ceased growth due to a lack of bacteria.

Clade-U protists grew on bacteriophage lysate retained by a 100 kD cut-off filter but not on the flow-through. Thus, low-molecular weight compounds in the lysate were not sufficient for growth, but proteins, viral particles, or aggregates from lysed bacteria larger than 100 kD and smaller than 0.45 μm were sufficient. The Clade-U protists also grew on lysates generated by infecting *Mycobacterium smegmatis* with Actinophage “Dulcie” (16, 17). Light and scanning electron microscopy (SEM) micrographs confirmed that little to no bacteria were present when these organisms were grown in phage lysates. Recent papers document that some protists can consume viruses under laboratory conditions and in natural environments (18–20). However, viral lysates have rarely, if ever, been used to isolate and propagate protist species, and this technique may allow for the culturing of some recalcitrant protists.

Four pure cultures identical in morphology and 18S rRNA gene sequence were independently propagated from single Clade-U cells. One of these subcultures was used in the studies described here, and the organism was named *N. vorax* for its unusually voracious feeding behavior and the neuron-like morphological appearance of some growth stages.

### Molecular phylogeny

**Fig 1:**
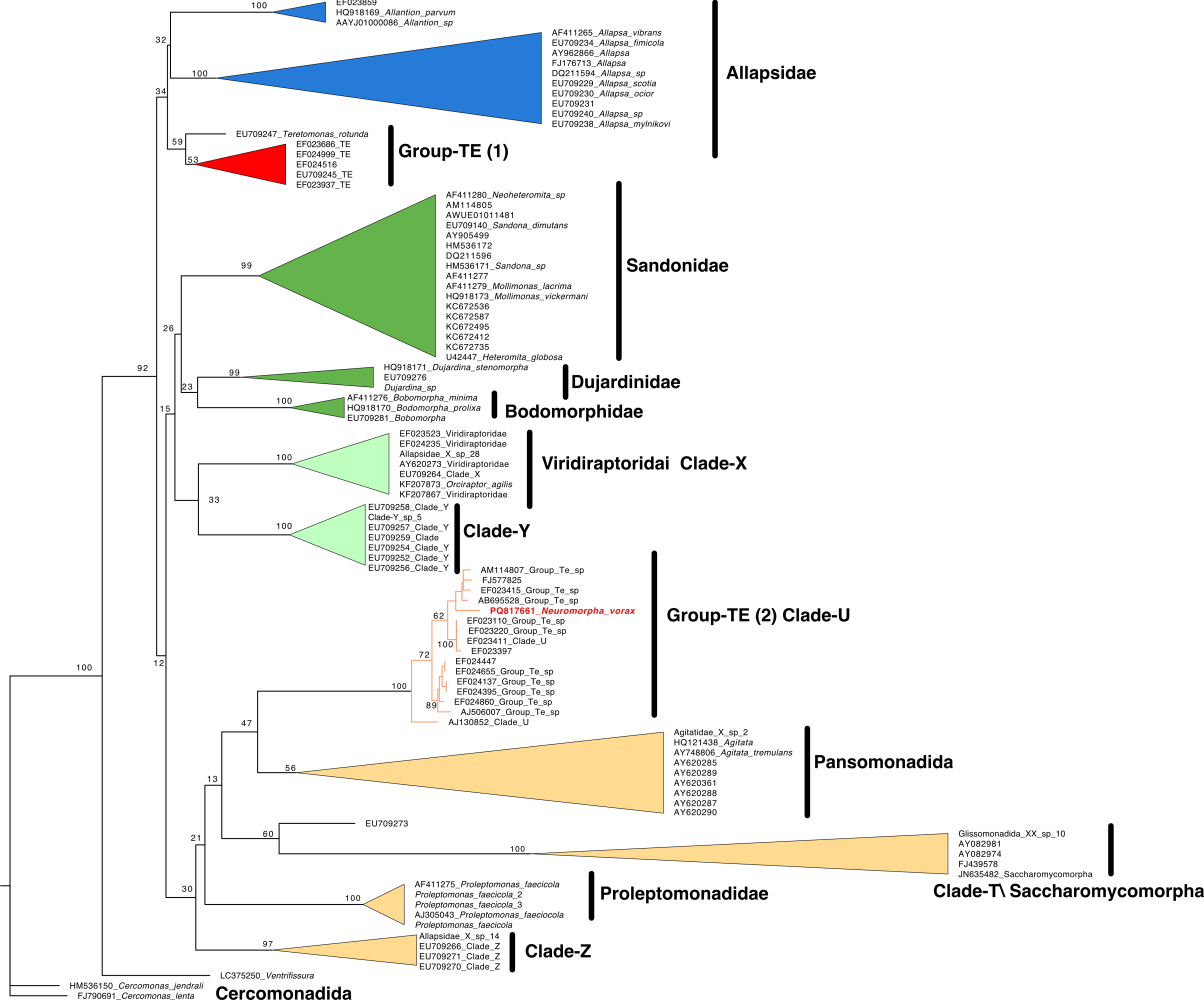
Phylogeny of Glissomonadida and related organisms. Pictured is the best maximum likelihood tree generated by IQ-TREE 2 based on a structural alignment of 18S gene sequences, as detailed in Materials and Methods. Sequences identified as Group-TE in the literature but that clustered with clade U sequences are denoted as “Group-TE/Clade-U” in the diagram. *N. vorax* is shown with red font. Support values are real bootstrap values based on 100 replicates. A detailed version of this tree is in Fig. S4 in the supplemental material.

The 18S rRNA gene sequence of *N. vorax* and a group of 99 18S rRNA genes from Glissomonadida and closely related groups were aligned using 18S rRNA structural information, and this was used to generate a maximum-likelihood tree (Fig. 1; Data set S1). The 18S sequences denoted as “Group-TE” in GenBank and in the curated PR2 database fell into two well-separated clades. One contained four sequences belonging to the Group-TE clade initially recognized by Howe et al. in papers describing the order Glissomonadida (9, 10). In Fig. 1, this clade clusters with 34% bootstrap support with Allapsidae and *Teretomonas,* as was shown previously (9, 10, 12, 13). *N. vorax* and the other 10 “Group-TE” sequences cluster with sequences in Clade-U of the Glissomonadida with high confidence (100% bootstrap support). This cluster is a sister to the Pansomonadida clade containing *Agitata* sp. and related organisms but with low bootstrap support of 47%. These are part of a larger clade that includes Clade-Z, Proleptomonadidae, and Clade-T/*Saccharomycomorpha* that have been noted by others (9, 10, 12, 13) but again with a low support value of 30%.

### Clade-U/TE organisms in the rhizosphere

Many papers referencing Group-TE as an important member of soil or rhizosphere communities are referring to members of the Clade-U/*Neuromorpha* cluster. Clade-U ASVs almost always identified as belonging to “Group-TE” are often some of the most abundant protist ASVs in environmental surveys of soil, and they are often enriched in rhizosphere samples relative to nearby bulk soil. Taerum et al. identified two Clade-U (“TE”) ASVs that were enriched in the core protist microbiome of field-grown maize (7). These (P07 and P08) were enriched in the rhizosphere 10- and 30-fold, respectively, compared to nearby bulk soil. Clade-U (“TE”) ASVs are also abundant core members of the wheat rhizosphere microbiome samples collected in Europe and Africa, as well as soil-grown lettuce, potato, tomato, and *Arabidopsis* from Germany and the US (5, 6, 15). Simonin et al. identified two Clade-U (TE”) ASVs in the core rhizosphere microbiome of wheat grown at two sites in Europe and two sites in Africa. One of these was the most abundant and prevalent protist in the core rhizosphere microbiome associated with wheat (6). Sapp et al. (5) identified five operational taxonomic units (numbers 12, 14, 106, 291, and 1427) that were rhizosphere-enriched in soil-grown *Arabidopsis thaliana*. These clustered with Clade-U sequences (Fig. 1; Data set S2).

The PR2 v5.0.1 database, which is a curated collection of 18S gene sequences focused on protists, contains 28 sequences identified as belonging to Group-TE (21). Twenty-four of these sequences group with Clade-U, and four cluster with Group-TE as defined by Howe et al. (9, 10). Most of the 24 Clade-U sequences were derived from soil or plant-associated (22–25). However, some were isolated from freshwater aquatic environments, such as microbial mats, lake water, or drinking water (26–29), indicating that Clade-U may be common in aquatic systems, too. These sequences, their associated data, and alignments are in Data set S2; Fig. S3.

### Life cycle of *N. vorax*

**Fig 2:**
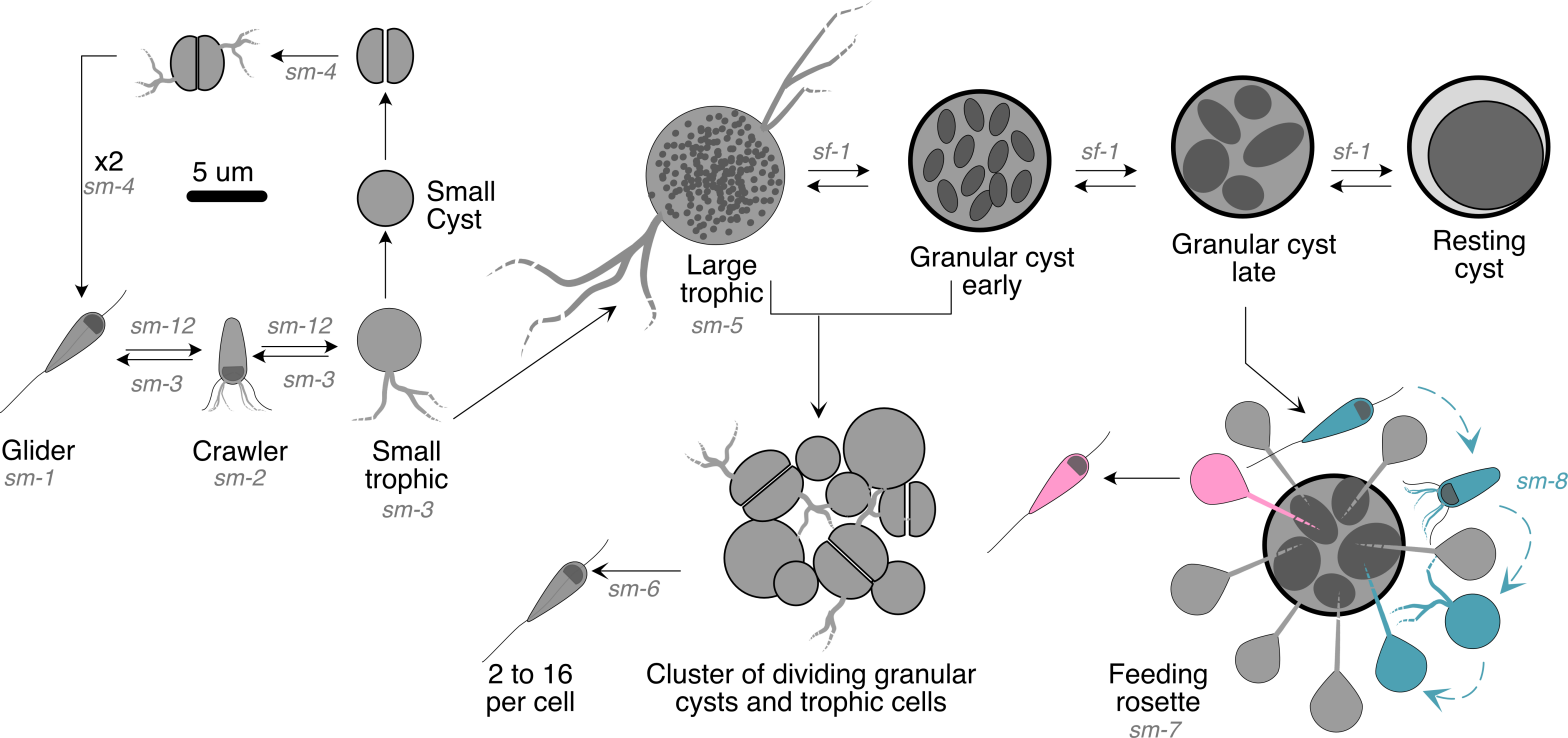
Life cycle of *N. vorax*. Various morphologies and transitions between those morphologies are outlined. Arrows indicate transitions that have been documented by time-lapse imaging or still images. Letters on the arrows indicate the supplemental movies (*sm-x*) and supplemental figures (*sf-1*) that document the transitions. The 5 mm scale bar shows scale for all the morphotypes in the figure. Cyan protists indicate how gliders enter the feeding rosette, and magenta protists indicate how they leave the rosette.

Culturing *N. vorax* with bacteriophage lysates eliminated visual interference by bacteria or other organisms and revealed a complex life cycle. This included a typical Glissomonad-like gliding form, as well as a crawling form, several trophic forms, distinct types of cysts, clusters of dividing cells, and communal and cannibalistic behavior (Fig. 2).

**Fig 3:**
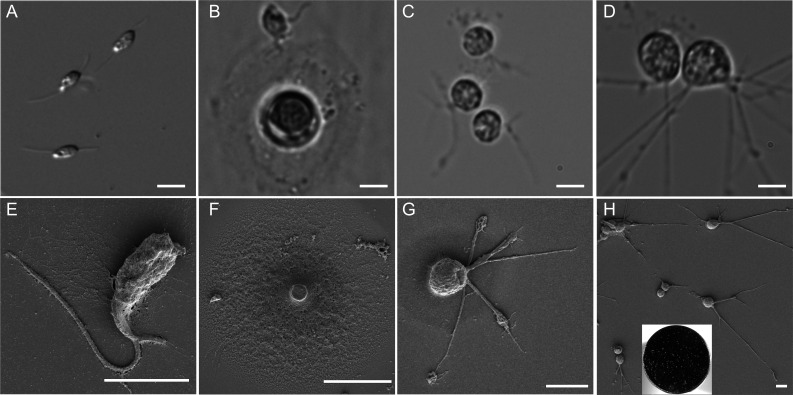
Basic forms of *N. vorax*. (**A**) Biflagellate gliding form. (**B**) Crawling (top) form interacting with the extracellular material excreted during the formation of a resting cyst (middle). (**C**) Small trophic cells on the bottom of the culture dish. (**D**) Larger trophic cells on the bottom of a culture dish. (**E**) Gliding form transitioning to crawler. (**F**) Large resting cyst. (**G**) Small trophic cell. (**H**) Large neuron-like trophic cells. Inset shows the designated hapantotype SEM stub3; white spots are clusters of *N. vorax* of various morphotypes. Panel A is a DIC image; panels B–D are bright-field images; and panels E–H are scanning electron micrographs. Scale bars indicate 5 mm. Additional SEM micrographs are in Fig. S2.

**Fig 4:**
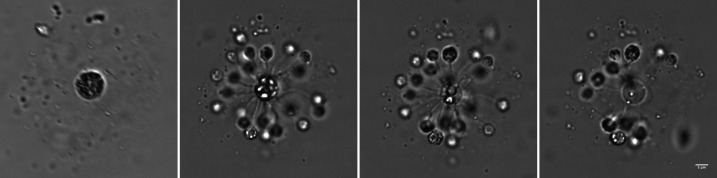
Feeding rosette formed by *N. vorax*. (**A**) Late-stage granulated cyst that was fed upon in the feeding rosette. This image was taken ~24 h before the rosette itself was imaged. (**B–D**) Feeding rosette consisting of the central cyst and about 25 small trophozoites. Note that the large refractile granules in the central cyst get smaller over time due to feeding and eventually disappear. Time lapse movie of this rosette is Movie S7. Scale of all panels is the same, and the scale bar indicates 5 mm.

#### Taxonomy (*N. vorax*)

Phylum Cercozoa Cavalier-Smith 1998

Class Sarcomonadea Cavalier-Smith 1993

Order Glissomonadida Howe, Bass, Vickerman Chao, and Cavalier-Smith, 2009

### Genus

*Neuromorpha* nov. gen. (urn:lsid:zoobank.org:act:5C12DABA-051D-46C7-95F7-073082FBEFC6).

### Genus description

Biflagellate gliders 3–8 µm long; feeding forms can vary from 5 to 15 µm and have thin non-anastomosing pseudopods; and can form large (10 µm) resting cysts that adhere to the substrate and are surrounded by a fibrous matrix.

### Genus etymology

Genus name refers to the neuron-like appearance of large trophic form.

### Type species

*N. vorax*.

### Species

*N. vorax* nov. sp. (urn:lsid:zoobank.org:act:8DCC5AF7-00FA-4BC4-B040-82668A32202E).

### Species etymology

*vorax* because of its voracious feeding habits.

### Collection site

*Zea mays* root rhizosphere from plants grown at the Connecticut Agricultural Experimental Station Lockwood Farm in July 2021.

### Type material

Fixed, dehydrated, and metal-sputtered SEM sample. The hapantotype is the collection of clonal cells grown on a coverslip and prepared as SEM stub 3 (Fig. 2H). The hapantotype specimen is deposited in the University of Connecticut Natural History Collections: SEM hapantotype accession number INV-49193.

### Type sequence

GenBank accession number: PQ817661

### Species description

Assumes a variety of forms as described below (Fig. 2 to 4).

### Gliding form

Gliding forms are biflagellate, and the cell body is teardrop-shaped and 3–9 µm in length depending on the availability of food. Gliding is smooth with no noted jerking, vibration, or other abrupt movements of the cell body. The anterior flagellum is approximately 1.2 times the length of the cell body when fully extended and pointed forward; the posterior flagellum is approximately two times the length of the cell body and trailed behind and in contact with the substratum during gliding (Fig. 3A and E; Movie S1).

### Crawling form

Gliding *N. vorax* can slow down, grow pseudopodia, and become loosely affixed to the substratum or to films, cysts, or aggregates in the growth medium (Fig. 3B). These then use flagella and pseudopods to crawl on the object to which they are attached. Crawlers could then either retract the pseudopods and transition back to gliding or retract their flagella and remain attached to the substrate as small amoeboid trophozoites (Movie S2).

### Small trophozoites

Small trophozoites have round cell bodies that were 4.6 +/− 0.8 μm in diameter. These have thin branched filopodia that most often emanate from one spot of the cell body. Filopodia contain enlarged regions that move in both directions along the length of the filament (Fig. 3C and G). These small trophic forms can transition back to gliders (Movie S3) or grow and form large granulated trophozoites. The small trophozoite can also form a small division cyst that undergoes binary division, resulting in two smaller trophozoites that become gliders (Movie S4).

### Large granular trophozoites

Small trophozoites can enlarge in size and become granulated. This occurs most frequently when grown in high lysate concentrations. Large trophozoites often have very long filopodia and resembled neurons (Fig. 3D and H). Granules can be seen moving along the filopodia in both directions (Movie S5). When grown in SES with phage lysate, no obvious particles were taken up or moved by the filopodia, suggesting that submicroscopic particles are being used as the food source.

### Granular cysts

Granular cysts form when granulated trophozoites retract their filopodia. Internal granules in large trophozoites merge into larger refractile granules. These cyst-like cells typically had four fates: (i) they remained unchanged; (ii) they divided and gave rise to four or more gliders; (iii) they grew and divided, giving clusters of cells (Movie S6); or (iv) the granules consolidated into one large granule, and the cell became a large resting cyst with a fried egg appearance (Fig. S1).

### Large resting cysts

These are 10.0 +/− 2.0 μm in diameter and contain a single, large, refractive granule surrounded by a circular matrix of extracellular material (Fig. 3B and F). This form is resistant to desiccation and can become active after rehydration, whereupon the large refractile granule separates into smaller granules. At this stage, they appear and behave like granular cysts.

### Small cysts

Small spherical cyst-like cells, which are 4.0 +/− 0.4 μm in diameter, appear in late-stage cultures when *N. vorax* is grown with low levels of phage lysate (10^7^ PFU/mL). Upon reactivation, small cysts divide and then develop in gliding forms (Movie S4).

### Feeding rosettes

Feeding rosettes form when gliding cells detect a late-stage granular cyst and attach as small trophozoites following a short intermediate stage as crawlers (Fig. 4; Movies S7 and S8). These small trophozoites are attached with a filament 10 to 15 μm long that is likely to be bundled filopodia. Filaments penetrated the outer layer of the cysts and embedded in the large granules contained in the cysts. These granules slowly disappear; presumably, they are consumed by the attached trophozoites. When the granules in the cyst are gone, the trophozoites convert back to gliders and swim away. The formation of these rosettes is relatively rare, but they were observed in different cultures at different times. Movie S9 shows a related example in which a single small trophozoite with a long filament consumes an aggregate or cyst at the edge of a submerged coverslip. Another cannibalistic behavior is shown in Movie S10 where gliders recognize a broken filopodium from a large trophic cell, then convert to small trophic cells and consume it.

## DISCUSSION

Sequences designated as belonging to the clade “Group-TE” of the Glissomonadida were first described by Howe et al. (9, 10). Since then, the Group-TE designation has been given to many sequences from environmental 18S rDNA surveys. The organisms from which these sequences derived have been noted as being widespread in terrestrial and freshwater ecosystems. They were often enriched and abundant in rhizosphere samples from around the world, indicating that they are well adapted to this niche. While it is not yet clear why *N. vorax* is enriched in rhizospheres, it may be enriched because its food source, phage, and/or lysed bacteria are also enriched in the rhizosphere. Alternatively, like Clade-X glissomonads, which can degrade agal cell walls and extract cytoplasm (13), *N. vorax* may interact directly with root cells. Either type of interaction in the rhizosphere could be either beneficial or detrimental to host plants.

Alignment of the 18S rRNA gene of *N. vorax* with those other glissomonads and related organisms showed that *N. vorax* and most sequences designated as Group-TE, including most of those known to be rhizosphere-enriched, clustered with sequences designated as Clade-U by Howe et al. This clade and the clade designated as “Group-TE” by Howe et al. are clearly distinct in the analyses presented here (Fig. 1), and a visual inspection of the alignments clearly shows that Group-TE and Clade-U (“TE”) have quite different 18S variable regions (Fig. S3; Data set S2). These organisms had not been cultured either through targeted efforts (30) or in research programs designed to isolate and characterize members of the Glissomonadida and their close relatives (9, 10, 31, 32). This led to the idea that the growth of these organisms perhaps required special conditions or partner organisms for growth.

We found that N. *vorax* was easy to grow and maintain in a standard soil-extract buffer supplemented with bacteriophage lysates from the soil bacteria *S. meliloti or M. smegmatis. N. vorax* did not grow on low-molecular weight compounds, such as proteins, lipids, or other small molecules that passed through a filter with 100 kD, but did grow on phage particles and larger bacterial debris retained by the filter.

When grown on less concentrated N3 lysates (~10^7^ PFU/mL), *N. vorax* was seen mostly as gliders, crawlers, and cysts. Small trophozoites were seen, but they were relatively rare and likely a quick transitional state on the way to small cysts. When high-titer lysates (~10^9^ PFU/mL) were used, the more complex *N. vorax* life cycle was revealed. Food, when abundant, allowed trophozoites to grow quite large and remain in that state for an extended period of time. These would then grow, divide, and give rise to gliders that could search for and colonize new areas for feeding and replication. This complex life cycle seen with high-titer lysates included cells with a variety of morphologies and behaviors not previously associated with members of the Glissomonadida. These included large trophozoites with multiple long filopodia and large resting cysts that were substrate-attached and resembled fried eggs. *N. vorax* displayed communal and cannibalistic feeding behaviors. It formed feeding rosettes, which consisted of a large granulated cyst at the center, ringed by small trophozoites that each consumed the contents of the central cyst through a long filament that was likely bundled filopodia. After the contents of the central cyst were drained by the trophozoites, they transformed back to gliders and swam away. This type of communal behavior may require signaling between the central cyst and the trophozoites that are attached to it. Alternatively, the feeding trophozoites may signal to motile cells, which then join the rosette. Related behaviors also suggested signaling between organisms. For example, gliding forms transitioned to crawlers, which interacted with the surface of resting cysts, with other crawlers, or with pseudopods that broke from or were ejected by large trophozoites (Movie S10).

The work outlined here has implications for culturing and characterizing other small hard-to-grow protists. Given the small size of gliding forms of many glissomonads, it is reasonable to suggest that these too may be culturable on phage lysates. This may allow the isolation and culturing of members from groups that have yet to be cultured (Group-TE, Clade-Y, and Clade-Z). In addition, given an abundant and readily consumed food source, such as bacteriophage lysate, and a clear unimpeded microscopic view of cell morphology and behavior, other species of glissomonads may be found to have life cycles and behaviors that are more complex than is currently appreciated. Hard-to-culture protists outside the Glissomonadida could be easier to culture and characterize using similar methods. It should be noted that pure phage lysates, *per se*, were not what allowed *N. vorax* to exhibit multiple morphologies and a complex life cycle. We observed *N. vorax* participating in feeding rosette behavior in its culture with *Paratetramitus* (see Movie S11). Lastly, the work described here shows that the products of viral activity, viral particles and cellular debris, can be used for the growth of eukaryotes. This suggests that viral activity in complex and crowded environments may directly support the growth (and not just death) of fellow community members.

## MATERIALS AND METHODS

### Enrichment of Group-TE protists and detection by nested PCR

*Z. mays* roots harvested from Connecticut Agricultural Experimental Station Lockwood Farm in July 2021 were saturated with sterile water, and after 7 days, the water was collected; 100 mL samples were put into 24-well microtiter plates containing 1 mL of SES buffer (SI Appendix); and heat-killed *Escherichia coli* strain DH5a cells were added to give a final OD_595_ of 0.005 (7). Following 4 weeks of incubation, these wells were tested for the presence of Clade-U protists using a high-sensitivity nested PCR approach. The first round of PCR amplified near full-length eukaryotic 18S amplicons. This was done with Promega GoTaq Master Mix (Madison, Wisconsin, USA) using primers EukBR (5-'TGATCCTTCTGCAGGTTCACCTA-3′) and 18SFU (5′-ATGCTTGTCTCAAAGGRYTAAGCCATG-3′). The PCR parameters were initial denaturation at 95°C for 5 min, followed by 35 cycles of 95°C for 30″, 60°C for 60″, and 72°C for 90″. The products were gel-purified following electrophoresis on a 1% agarose gel. Next, 1–5 µL of the purified products was used in a second round of amplification with Promega GoTaq Master Mix using primers Clade-U_F (5′-CTGRCGAAACTGCTAGCTG 3′) and Clade-U_R (5′-TTGTGTTGCCACAAGAG GCC-3′). The PCR parameters were initial denaturation at 95°C for 5 min, followed by 35 cycles of 95°C for 30″, 64°C for 60″, and 72°C for 90″. The products were gel-purified using the following electrophoresis on 1% agarose gel and sent to Azenta (Chelmsford, Massachusetts, USA) for Sanger sequencing. Wells that were positive for Clade-U by this test went through a series of 1:10 to 1:100 dilutions into wells with 1 mL of fresh SES and heat-killed *E. coli* DH5a at the concentration listed above. Eventually, wells containing only Clade-U and a *Paratetramitus* sp., as determined by community 18S sequencing, were isolated and propagated.

### Purification, growth, and maintenance with phage lysates

Dilution and single-cell isolations to separate Clade-U protists from *Paratetramitus* were unsuccessful probably because the Clade-U protists were removed from their food source. Various bacteria (some of which were spore formers) were isolated from the well and tested to see if they could support the growth of the Clade-U protists—none did. These bacteria and their 16S rDNA sequences are presented in Fig. S1 in the supplemental material. Accordingly, 100 μL of a 10^10^ PFU/mL phage lysate made by growing bacteriophage N3 on *Sinorhizobium meliloti* (33) was added to fresh wells made by diluting a 1 month-old mixed culture of the Clade-U protists, *Paratetramitus* sp., and resident bacteria. These phage lysate additions promoted the growth of Clade-U protists and completely suppressed the growth of *Paratetramitus* and the bacteria in the well. Purified lines of Clade-U generated by isolating single cells from an initial lysate grown well were maintained in either 24-well plates containing 1 mL of SES plus 10^7^–10^8^ PFU of N3 lysate or in 25 mm^2^ plastic tissue culture flasks containing 5 mL of SES plus 5 × 10^7^ to 5 × 10^8^ PFU of N3 lysate. Sequences of the full-length 18S rRNA gene of all four lines were identical.

### Phage lysates

*S. meliloti* strain Rm1021 was grown in TY medium with 250 mg/mL streptomycin for 48 h. Next, 200 mL of these cells was added to 13 × 100 mm glass tubes with 100 mL of a N3 bacteriophage stock that had been diluted 10^−2^- to 10^−5^-fold in TY. After 20 min, 5 mL of TY soft agar (at ~45°C) was added to each tube, and the mixture was poured onto room-temperature TY plates. Following overnight incubation at 30°C, plates with “lacy” or near-confluent lysis were overlaid with 5 mL of SM buffer (SI Appendix) overnight. The SM overlays were pooled (~20–30 mL), filter-sterilized with a 0.45 mm syringe filter, and dialyzed (12–14 kD cut-off) twice for 12 h against 500 mL of SM buffer. This was then resterilized by filtration as before and stored at 4°C. Lysates typically contained 10^8^–10^9^ PFU/mL.

### Microscopy

Protists were grown in 24-well microtiter dishes, and imaging was done through the bottom of the wells on a Nikon TE300 inverted microscope with extra-long working distance objectives. Images and time-lapse videos were captured with a Basler acA2040 monochrome CMOS camera (2,056 × 1,540 px, 3.5 µm pixel size). The microscope stage, camera, and LED illumination were controlled with Micromanager v. 2.0 gamma (34).

### Sequencing

Genomic DNA was isolated from 0.5 mL samples of *N. vorax* grown in 1.2 mL of SES + N3 (10^8^ PFU/mL) using an MP Biomedicals FastDNA Spin Kit for Soil (San Diego, California, USA). Near full-length 18S rDNA was amplified with primers EukBR and 18SFU using NEB Phusion HF Polymerase Master Mix (Ipswich, MA, USA). The PCR parameters were initial denaturation at 96°C for 60″, followed by 35 cycles of 94°C for 20″, 68°C for 30″, and 72°C for 90″. Products were run on 1% agarose gel and purified from the gel, and PCR product sequencing was performed by Plasmidsaurus using Oxford Nanopore Technology (Eugene, Oregon, USA).

### Phylogenetic analysis

The 18S gene sequence of *N. vorax* was aligned with 100 other near-full-length sequences of glissomonads from the PR2 database and the National Center for Biotechnology Information RefSeq using the software package ssu-align-v0.1.1 (35, 36). This package aligned our 99 representative 18S rDNA sequences to a database of 18S sequences from eukaryotes based on sequence and secondary structures. A total of 2,349 columns of information were present in the aligned sequences. Secondary structure, consensus, and insert designations are included in the Stockholm format output of ssu-align. Multiple stepwise masking of the ssu-align alignment was done using the ssu-mask feature of the ssu-align package and trimal (37). The scripts for these and the starting alignment files are in Fig. S1 in the supplemental material and Data set S1.

The software package IQ-TREE 2 (38) was used to generate ultrafast-bootstrap phylogenetic trees using the “-B 1000” argument. The substitution model, state frequency, and rate heterogeneity type were selected by IQ-TREE 2 (39, 40). This initial work showed that masking affected the reproducibility of the trees Data set S3. Most trees were similar, with Clade-U and Pansomonadida as sister groups. The trimal “trimal -in $FASTA -out $OUT -gt .70” resulted in the tree with the best overall ultrafast support values. Standard bootstrap is a more conservative and reliable indicator of branch support than the support values used in the faster initial tree building trials. Because of this, we regenerated the trimal “-gt .70” tree and other trees with good overall ultrafast bootstrap support using IQ-TREE 2 with normal bootstrap values (-b 100). Fig. 1 was generated with IQ-TREE using the trimal “-gt .70” alignment. IQ-TREE was allowed to select the best substitution model, state frequency, and rate heterogeneity type (TIM + F + I + R4) for the trimal “-gt .70” alignment. Scripts for ssu-align, ssu-mask, trimal, and IQ-TREE 2 are provided in SI Appendix. The original unaligned sequences, the unmasked alignment in fasta and Stockholm formats, and the “trimal -gt .70” masked alignment in fasta format are provided in Data set S1.

## Data Availability

The 18S rRNA gene sequence is deposited under GenBank accession number PQ817661. Descriptions of the genus Neuromorpha and the species Neuromorpha vorax can be found at ZooBank: Neuromorpha nov. gen. (urn:lsid:zoobank.org:act:5C12DABA-051D-46C7-95F7-073082FBEFC6) and Neuromorpha vorax nov. sp. (urn:lsid:zoobank.org:act:8DCC5AF7-00FA-4BC4-B040-82668A32202E). 18S rRNA gene sequences, alignments, and data on bootstrap values vs alignment masking are included in the supplementary material. Scripts and sequence data used to make the phylogenetic trees are included in the supplementary materials.

## SUPPLEMENTAL MATERIAL

Data set S1 mbio.00848-25-s0001.txt 18S rRNA structural alignments.

Data set S2 mbio.00848-25-s0002.xlsx Clade-U and TE information.

Data set S3 mbio.00848-25-s0003.xlsx Masking vs. clade support.

Supplemental material mbio.00848-25-s0004.docx Supplemental figures, legends for data sets and movies, and text.

Movies S1–S3 mbio.00848-25-s0005.mov Gliders, crawler on particle, and small trophic cell converting to glider.

Movies S4–S6 mbio.00848-25-s0006.mov Division cyst, large trophic cell, and cluster releasing gliders.

Movies S7–S11 mbio.00848-25-s0007.mov Feeding rosette, addition to feeding rosette, trophozoites with aggregate, pseudopod interactions, and feeding rosette in complex culture.

ASM does not own the copyrights to Supplemental Material that may be linked to, or accessed through, an article. The authors have granted ASM a non-exclusive, world-wide license to publish the Supplemental Material files. Please contact the corresponding author directly for reuse.
