# Supplementary material for: Neuromorpha vorax: a previously unculturable cosmopolitan protist with an unexpectedly complex life cycle belonging to Glissomonadida Clade-U/Group-TE
Source: mBio. 2025 May 30;16(7):e00848-25. doi: 10.1128/mbio.00848-25 (PMC12239558; doi:10.1128/mbio.00848-25)
Supplement: Supplemental material — Supplemental figures, legends for data sets and movies, and text. [file mbio.00848-25-s0004.docx]

***Neuromorpha vorax*: characterization of a cosmopolitan protist with an unexpectedly complex life-cycle belonging to Glissomonadida Clade-U/Group-TE**

**Authors:** Gabrielle Corso^a,^, Lindsay R. Triplett^b^ and Daniel J. Gage^a*^

**Corresponding author:** Daniel J. Gage

**Email:** [daniel.gage@uconn.edu](mailto:xxxxx@xxxx.xxx)

**This PDF file includes:**

Figures S1 to S4

Legends for Datasets S1 to S3

Legends for Movies S1 to S12

Supporting text

**Other supporting materials for this manuscript include the following:**

Datasets S1 to S3

Movies S1 to S12

**Supplemental Figures**

*
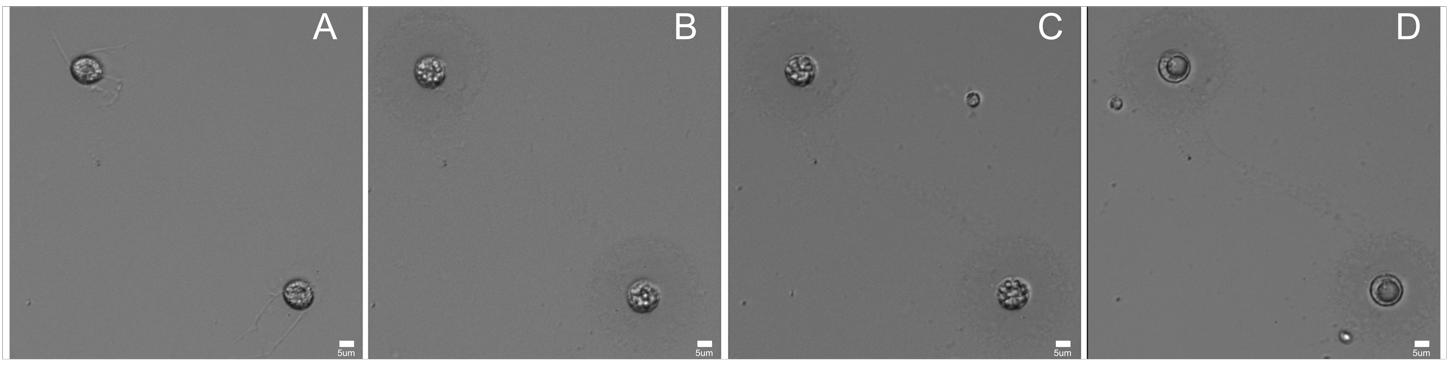
*

**Figure S1. Granular cysts developing to resting cysts.** Montage of two large trophic cells developing into two large resting cysts. A) Two large trophic cells. B) Early stage granular cysts. C) Late stage granular cysts. D) Resting cysts. Scale bars are 5 mm.

**
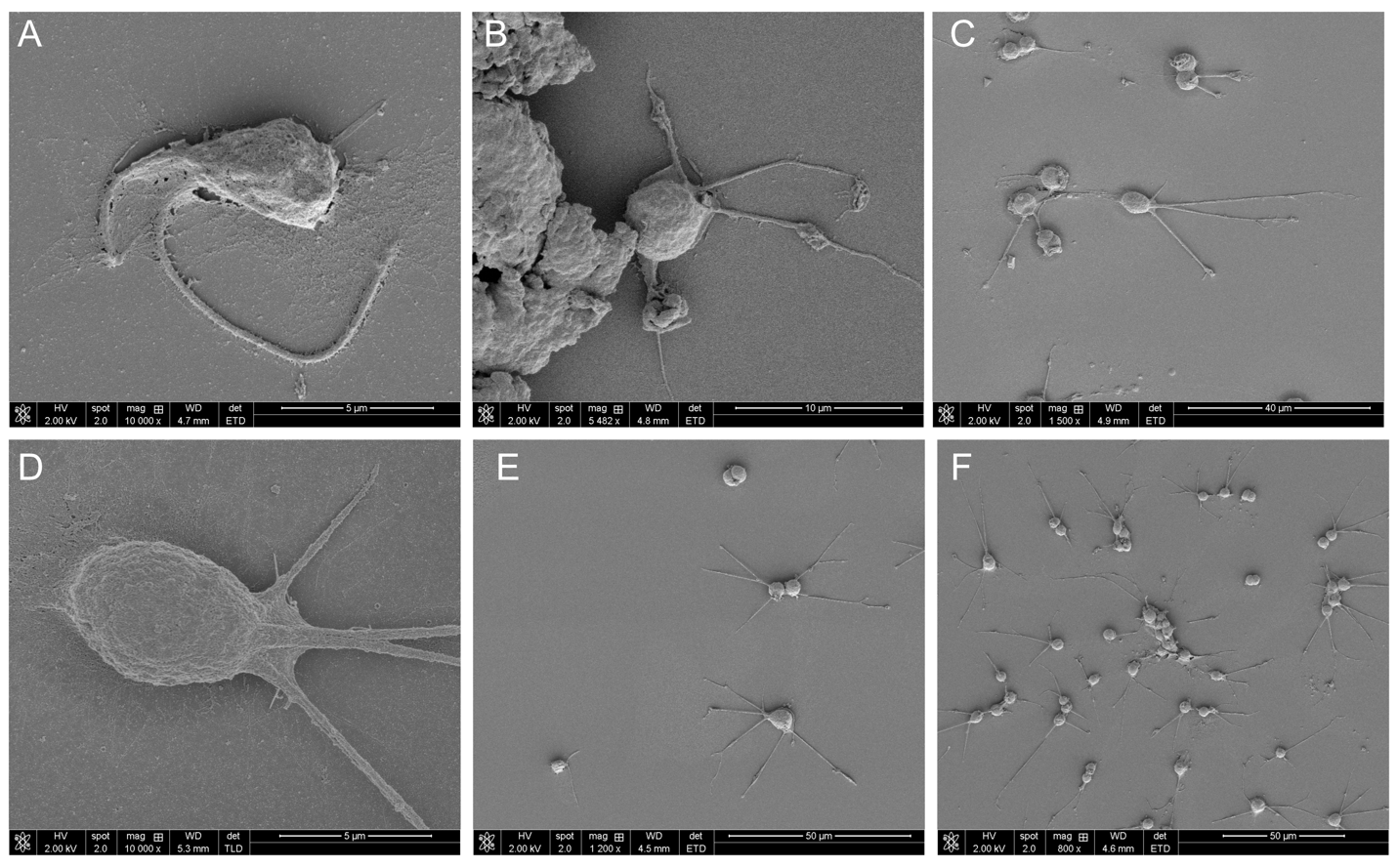
**

**Figure S2. Additional SEM micrographs of *N. vorax.*** A) Gliding form with two visible flagella. B) Small trophic cell with four visible filopodia. C-F) Combinations of small and larger feeders. Other forms can be seen such as a glider in the lower left of E) and a dividing cell in upper-central portion of that same panel. D) is a close up of a larger feeder shown in the middle of panel C).


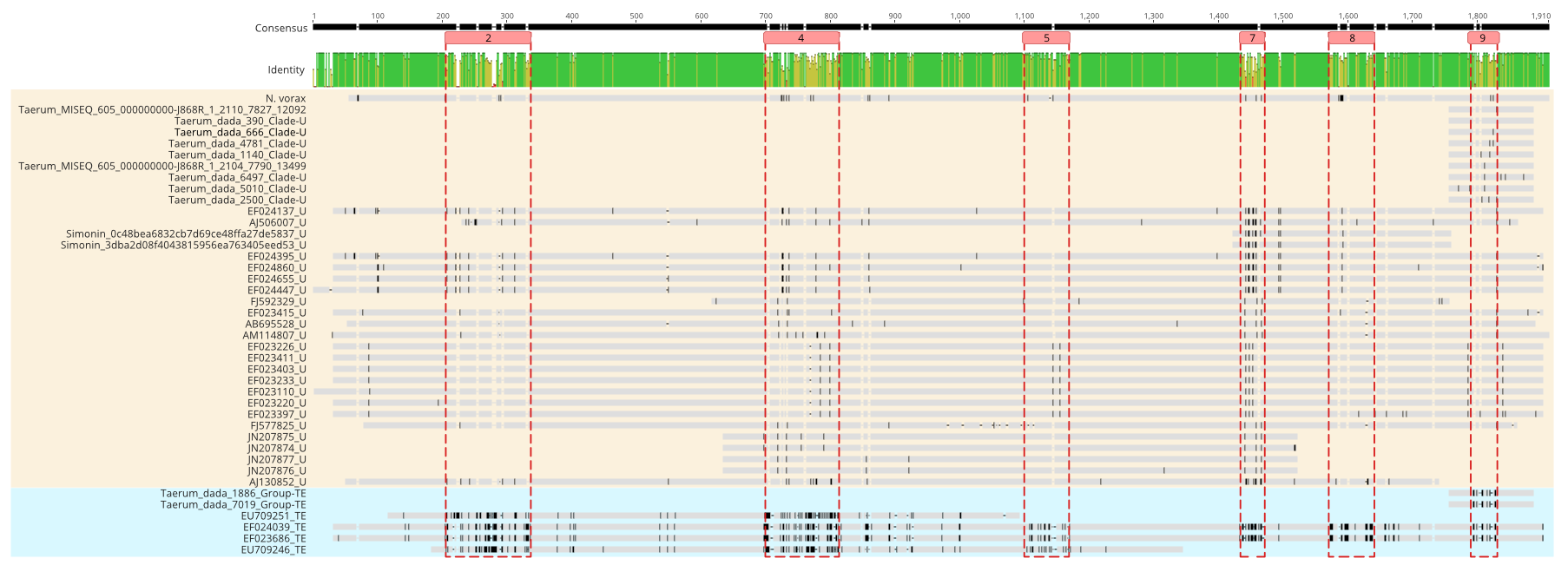


**Figure S3. *Clade-U vs Group-TE alignment.*** Overview of alignments of 41 18S rRNA gene sequences. Vertical black lines show disagreements between sequences and the consensus sequence of the group. Clade U sequences are shown in tan, Group-TE as described by Howe et al. are in blue.

**Figure S4. Phylogenetic tree with details.** Detailed version of the phylogenetic tree shown in Fig. 1.

**Supplemental_Data Sets**

**Supplemental Dataset 1. 18S rRNA structural alignments.txt. Original unaligned fasta fiel,** unmasked alignment in fasta and Stockholm formats and the “trimal_out_gp70.fasta” masked alignment (used to generate Fog1), provided in a single file.

**Supplemental Dataset 2. Clade-U and TE information.xlsx.** A spreadsheet containing sequences, metadata and alignments of members of Group-TE and Clade-U\TE from the literature and from the PR2 database.

**Supplemental Dataset 3. Masking vs clade support.xlsx.** Analysis of how masking 18S structural alignments using ssu-mask affected bootstrap support between the Group TE and Clade-U and their adjacent clades.

***Supplemental Movies***

**Supplemental Movie 1. Gliders.** *N. vorax* gliders. Frames taken 0.1 sec apart, playback = 6fps = close to real-time.

**Supplemental Movie 2. Crawler on particle.** *N. vorax* crawler interacting with aa aggregate. Frames taken 10 sec apart, playback = 10fps = 100x real-time.

**Supplemental Movie 3. Small trophic cell converting to glider.** Small trophic form of *N. vorax* converting back to gliding form. Frames taken 10 sec apart, playback = 10fps = 100x real-time.

**Supplemental Movie 4. Division cyst.** Small reproductive cyst developing into two gliding forms. Frames taken 10 sec apart, playback = 20fps = 200x real-time. There is a gap about 90% of the way through the film, where the time lapse had stopped and was then restarted 25 minutes later.

**Supplemental Movie 5. Large trophic cell.** Large granular trophozoite feeding on phage lysate. Frames taken 2 sec apart, playback = 20fps = 40x real-time

**Supplemental Movie 6. Cluster releasing gliders.** The cluster of feeders on the bottom derived from a granulated cyst, similar to the other two cells in the movie. Cells in the cluster will convert from trophozoites with filopodia to cysts, which then release glider forms. Frames taken 10 sec apart, playback = 20fps = 200x real-time.

**Supplemental Movie 7. Feeding rosette.** Feeding rosette with ~25 small trophozoite forms of *N. vorax* feeding on a central *N. vorax* cyst. Frames taken 10 sec apart, playback = 20fps = 200x real-time.

**Supplemental Movie 8 Addition to feeding rosette addition.** Addition of a gliding cell to a feeding rosette. This cell transitions to a crawler and then adds to the rosette as a small trophozoite. Frames taken 10 sec apart, playback = 20fps = 200x real-time.

**Supplemental Movie 9 Small trophic cell consuming an aggregate.** A single small trophozoite with bundled filipodia consumes an aggregate at the edge of a coverslip. A second trophozoite adds to the aggregate near the bottom of the frame early in the movie. Frames taken 10 sec apart, playback = 20fps = 200x real-time.

**Supplemental Video 10 Pseudopod interactions.** Two gliders interacting with, and possibly consuming, a pseudopod that is broken, or ejected, from a large feeding trophozoite. Frames taken 10 sec apart, playback = 20fps = 200x real-time.

**Supplemental Video 11. Feeding rosette in complex culture.** A feeding rosette in the culture composed of various bacteria, Paratetramitus and N. vorax. A glider can be seen adding to bottom of the rosette about 1/3 of the way through the movie. Frames taken 10 sec apart, playback = 20fps = 200x real-time.

**Supplemental Methods**

***1) Recipes***

***A) SES (from CCAP (Culture Collection of Algae and Protozoa))***

| **Stock Solution** | **Ingredient** | **gr/liter** |
| --- | --- | --- |
| 1 | K_2_HPO_4_ | 1 |
| 2 | KNO_3_ | 1 |
| 3 | MgSO_4_•7H_2_O | 10 |

To make SES

| **Ingredient** | **ml per liter** |
| --- | --- |
| Stock solutions 1 ­ 3 | 20 |
| Soil extract (see below) | 100 |

Make to one liter and autoclave

**SE2 (Soil Extract 2) for freshwater and terrestrial protozoa**

Site selection for a good soil is very important and for most purposes a soil from undisturbed deciduous woodland is best. Sites to avoid are those showing obvious signs of man's activity and particular care should be taken to avoid areas where fertilizers, crop sprays or other toxic chemicals may have been used.

A rich loam with good crumb structure should be sought. Stones, roots and larger invertebrates should be removed during an initial sieving through a 1 cm mesh. The sieved soil should be spread to air dry and hand picked for smaller invertebrates and roots. It should be turned periodically and picked over again. When dry it may be sieved through a finer mesh (2­4 mm) or stored as it is prior to use.

**Making the extract**

Soil is prepared as above. 105 g of air­dried sieved soil and 660 ml of deionized water are placed in a 1 litre bottle and autoclaved once at 15 psi for 15 minutes, then again after 24 hours. The contents of the bottle are left to settle (usually for at least a week) and then the supernatant is decanted and filtered. The final pH should be 7.0 - 8.0.

-------------------

***B) TY***

| **Ingredient** | **g per liter** |
| --- | --- |
| Tryptone | 6 |
| Yeast extract | 3 |
| CaCl_2_ | .5 |

Make to one liter and autoclave

***C) SM buffer [Bonilla PeerJ 2016]***

| **Ingredient** | **per liter** |
| --- | --- |
| NaCl | 5.8 gr |
| MgSO4•7H2O | 2 gr |
| 1M Tris-HCl pH 7.4 | 50 ml |

Make to one liter and filter sterilize

***2) SSU-Align and IQ-tree2 scripts***

A) SSU-align and mask and trimal scripts

#!/bin/bash

############################################ SSU-ALIGN and MASK script is below

DIRNAME=ssu-align

FASTA=trial16.fasta

#

# #run ssu-align on the fasta file

# --dna (output alignments as DNA, default is RNA (even if input is DNA))

ssu-align --dna $FASTA $DIRNAME

###############################################

################################################

#run a variety of masks on the ssu-align output files, and save alignments as .afa files

# --stk2afa (convert Stockholm alignment(s) to aligned FASTA)

# --afa (output aligned FASTA (.afa) alignments, not Stockholm ones)

# --pf <x> (include columns w/<x> fraction of seqs w/prob >= --pt <y> [df: 0.95])

# --pt <y> (set probability threshold as <y> [default=0.95])

# --rfonly (exclude insert (gap in RF) columns only)

# --key-out <s> (add <s> to all output file names, before the suffix)

#default mask based on alignment

KEY="default_mask"

ssu-mask --dna --afa --key-out $KEY $DIRNAME

#set eukaryotic mask

KEY="set_mask"

ssu-mask --dna --afa --key-out $KEY -d $DIRNAME

KEY="rf_only_mask"

ssu-mask --dna --afa --key-out $KEY --rfonly $DIRNAME

#set eukaryotic mask

KEY="pf95_pt80_mask"

ssu-mask --dna --afa --key-out $KEY --pf .95 --pt .80 $DIRNAME

#set eukaryotic mask

KEY="pf90_pt90_mask"

ssu-mask --dna --afa --key-out $KEY --pf .90 --pt .90 $DIRNAME

#set eukaryotic mask

KEY="pf90_pt80_mask"

ssu-mask --dna --afa --key-out $KEY --pf .90 --pt .80 $DIRNAME

#set eukaryotic mask

KEY="pf85_pt90_mask"

ssu-mask --dna --afa --key-out $KEY --pf .85 --pt .90 $DIRNAME

#set eukaryotic mask

KEY="pf80_pt90_mask"

ssu-mask --dna --afa --key-out $KEY --pf .80 --pt .90 $DIRNAME

#set eukaryotic mask

KEY="pf75_pt90_mask"

ssu-mask --dna --afa --key-out $KEY --pf .75 --pt .90 $DIRNAME

#set eukaryotic mask

KEY="pf70_pt90_mask"

ssu-mask --dna --afa --key-out $KEY --pf .70 --pt .90 $DIRNAME

#set eukaryotic mask

KEY="pf60_pt90_mask"

ssu-mask --dna --afa --key-out $KEY --pf .60 --pt .90 $DIRNAME

#set eukaryotic mask

KEY="pf50_pt90_mask"

ssu-mask --dna --afa --key-out $KEY --pf .50 --pt .90 $DIRNAME

#set eukaryotic mask

KEY="pf50_pt80_mask"

ssu-mask --dna --afa --key-out $KEY --pf .50 --pt .80 $DIRNAME

#set eukaryotic mask

KEY="pf50_pt70_mask"

ssu-mask --dna --afa --key-out $KEY --pf .50 --pt .70 $DIRNAME

#set eukaryotic mask

KEY="pf50_pt60_mask"

ssu-mask --dna --afa --key-out $KEY --pf .50 --pt .60 $DIRNAME

#set eukaryotic mask

KEY="pf45_pt80_mask"

ssu-mask --dna --afa --key-out $KEY --pf .45 --pt .80 $DIRNAME

#set eukaryotic mask

KEY="pf40_pt80_mask"

ssu-mask --dna --afa --key-out $KEY --pf .40 --pt .80 $DIRNAME

#set eukaryotic mask

KEY="pf40_pt60_mask"

ssu-mask --dna --afa --key-out $KEY --pf .40 --pt .60 $DIRNAME

#set eukaryotic mask

KEY="pf40_pt70_mask"

ssu-mask --dna --afa --key-out $KEY --pf .40 --pt .70 $DIRNAME

--------------------------------

The Stockholm formated output of ssu-align " ssu-align.eukarya.stk" was converted to fasta format using the ssu-mask argument "--stk2fasta". That output " trial16_NoMasking.fasta" was used as input into the following trimal masking script:

#!/bin/bash

FASTA=trial16_NoMasking.fasta

#

# #run trimal on the aligned file

OUT=trimal_out_NoGaps.fasta

trimal -in $FASTA -out $OUT -nogaps

#

# #run trimal on the aligned file

OUT=trimal_out_NoAllGaps.fasta

trimal -in $FASTA -out $OUT -noallgaps

# #run trimal on the aligned file

OUT=trimal_out_GappyOut.fasta

trimal -in $FASTA -out $OUT -gappyout

# #run trimal on the aligned file

OUT=trimal_out_Strict.fasta

trimal -in $FASTA -out $OUT -strict

# #run trimal on the aligned file

OUT=trimal_out_StrictPlus.fasta

trimal -in $FASTA -out $OUT -strictplus

# #run trimal on the aligned file

OUT=trimal_out_Automated1.fasta

trimal -in $FASTA -out $OUT -automated1

# #run trimal on the aligned file

OUT=trimal_out_gp60.fasta

trimal -in $FASTA -out $OUT -gt .60

# #run trimal on the aligned file

OUT=trimal_out_gp70.fasta

trimal -in $FASTA -out $OUT -gt .70

# #run trimal on the aligned file

OUT=trimal_out_gp80.fasta

trimal -in $FASTA -out $OUT -gt .80

# #run trimal on the aligned file

OUT=trimal_out_gp90.fasta

trimal -in $FASTA -out $OUT -gt .90

# #run trimal on the aligned file

OUT=trimal_out_gp95.fasta

trimal -in $FASTA -out $OUT -gt .95

#run trimal on the aligned file

OUT=trimal_out_gp50.fasta

trimal -in $FASTA -out $OUT -gt .50

# #run trimal on the aligned file

OUT=trimal_out_gp40.fasta

trimal -in $FASTA -out $OUT -gt .40

# #run trimal on the aligned file

OUT=trimal_out_gp30.fasta

trimal -in $FASTA -out $OUT -gt .30

# #run trimal on the aligned file

OUT=trimal_out_gp20.fasta

trimal -in $FASTA -out $OUT -gt .20

# #run trimal on the aligned file

OUT=trimal_out_gp10.fasta

trimal -in $FASTA -out $OUT -gt .10

# #run trimal on the aligned file

OUT=trimal_out_gp05.fasta

trimal -in $FASTA -out $OUT -gt .05

B) IQ-tree2 script

###################################### IQ-tree script is below #############

#!/bin/bash

#make ultrafast boot trees for quick overview of trees

DIR=alignment_location

# cd to dir with .fasta alignment files

# make trees of each .fasta file in a folder

cd $DIR

# -m TESTNEW (test models, select best, construct trees)

### CAN ALSO SELECT A PARTICULAR MODEL. FOR EXAMPLE:

# -m GTR+R4+F

# -B 1000 (ultrafast bootstrap 1000 reps)

# --nstop 200 (Number of unsuccessful iterations to stop during tree search (default: 100))

# --sprrad 12 (Specify SPR radius for the initial parsimony tree search. DEFAULT: 6)

# --allnni (Perform more thorough NNI search (default: OFF))

# -ninit 500 (Number of initial parsimony trees (default: 100))

# -ntop 50 (Number of top initial trees (default: 20))

# --runs 10 (Number of indepedent runs (default: 1))

### CAN ALSO ADD EXTRA BRANCH SUPPPORT VALUES:

# --alrt 1000 (Parametric aLRT test (Anisimova and Gascuel 2006))

# --abayes (approximate Bayes test (Anisimova et al. 2011))

for FILE in *.fasta

do

iqtree2 -s $FILE -m TESTNEW -B 1000 -T AUTO -o FJ790691_Cercomonas_lenta --prefix $FILE --nstop 200 --sprrad 12 --allnni -ninit 500 -ntop 50

done

# FOR STANDARD BOOTSTRAP SUPPORT VALUES 100 trees (slower! but better)

for FILE in *.fasta

do

iqtree2 -s $FILE -m TESTNEW -b 100 -T AUTO -o FJ790691_Cercomonas_lenta --prefix trial15_realigned_out.rf_only.fasta --nstop 200 --sprrad 12 --allnni -ninit 500 -ntop 50

done

##########################################################################

C) Bacteria isolated from well with Clade-U protists and *Paratetramitus* and tested in single cell protists isolations

>BC1_isolate1_Priestia_megaterium

TTGGGCGTAAGCGCGCGCAGGCGGTTTCTTAAGTCTGATGTGAAAGCCCACGGCTCAACCGTGGAGGGTCATTGGAAACTGGGGAACTTGAGTGCAGAAGAGAAAAGCGGAATTCCACGTGTAGCGGTGAAATGCGTAGAGATGTGGAGGAACACCAGTGGCGAAGGCGGCTTTTTGGTCTGTAACTGACGCTGAGGCGCGAAAGCGTGGGGAGCAAACAGGATTAGATACCCTGGTAGTCCACGCCGTAAACGATGAGTGCTAAGTGTTAGAGGGTTTCCGCCCTTTAGTGCTGCAGCTAACGCATTAAGCACTCCGCCTGGGGAGTACGGTCGCAAGACTGAAACTCAAAGGAATTGACGGGGGCCCGCACAAGCGGTGGAGCATGTGGTTTAATTCGAAGCAACGCGAAGAACCTTACCAGGTCTTGACATCCTCTGACAACTCTAGAGATAGAGCGTTCCCCTTCGGGGGACAGAGTGACAGGTGGTGCATGGTTGTCGTCAGCTCGTGTCGTGAGATGTTGGGTTAAGTCCCGCAACGAGCGCAACCCTTGATCTTAGTTGCCAGCATTTAGTTGGGCACTCTAAGGTGACTGCCGGTGACAAACCGGAGGAAGGTGGGGATGACGTCAAATCATCATGCCCCTTATGACCTGGGCTACACACGTGCTA

>BC1__isolate2_Paenibacillus_glycanilyticus

ATTGGGCGTAAGCGCGCGCAGGCGGCCTTGTAAGTCTGTCGTTTAAACTCGGAGCTCAACTTCGAGTCGCGATGGAAACTGCAAAGCTTGAGTGCAGAAGAGGAAAGTGGAATTCCACGTGTAGCGGTGAAATGCGTAGAGATGTGGAGGAACACCAGTGGCGAAGGCGACTTTCTGGGCTGTAACTGACGCTGAGGCGCGAAAGCGTGGGGAGCAAACAGGATTAGATACCCTGGTAGTCCACGCCGTAAACGATGAATGCTAGGTGTTAGGGGTTTCGATACCCTTGGTGCCGAAGTTAACACATTAAGCATTCCGCCTGGGGAGTACGGTCGCAAGACTGAAACTCAAAGGAATTGACGGGGACCCGCACAAGCAGTGGAGTATGTGGTTTAATTCGAAGCAACGCGAAGAACCTTACCAGGTCTTGACATCCCTCTGACCGTCCTAGAGATAGGGCTTTCCTTCGGGACAGAGGAGACAGGTGGTGCATGGTTGTCGTCAGCTCGTGTCGTGAGATGTTGGGTTAAGTCCCGCAACGAGCGCAACCCTTGATCTTAGTTGCCAGCACTTTGGGTGGGCACTCTAGGATGACTGCCGGTGACAAACCGGAGGAAGGTGGGGATGACGTCAAATCATCATGCCCCTTATGACCTGGGCTACACACGTACTACAATGGCCGATACAA

>BC1__isolate3_Paenibacillus_pocheonensis

TGGGCGTAAGCGCGCGCAGGCGGTTAATTAAGTTGGGTGTTTAAGCCCGGGGCTCAACCCCGGTTCGCATCCAAAACTGGTTGACTTGAGTGTAGGAGAGGAAAGTGGAATTCCACGTGTAGCGGTGAAATGCGTAGAGATGTGGAGGAACACCAGTGGCGAAGGCGACTTTCTGGCCTATAACTGACGCTGAGGCGCGAAAGCGTGGGGAGCAAACAGGATTAGATACCCTGGTAGTCCACGCCGTAAACGATGCATACTAGGTGTTGGGGATTCGATTCCTCGGTGCCGAAGTTAACACAGTAAGTATGCCGCCTGGGGAGTACGCTCGCAAGAGTGAAACTCAAAGGAATTGACGGGGACCCGCACAAGCAGTGGAGTATGTGGTTTAATTCGAAGCAACGCGAAGAACCTTACCAGGTCTTGACATCCCTCTGACCGGTCTGGAGACAGGCCTTCCCTTCGGGGCAGAGGAGACAGGTGGTGCATGGTTGTCGTCAGCTCGTGTCGTGAGATGTTGGGTTAAGTCCCGCAACGAGCGCAACCCTTGATCTTAGTTGCCAGCACTTCGGGTGGGCACTCTAAGATGACTGCCGGTGACAAAACCGGAGGAAGGTGGGGATGACGTCAAATCATCATGCCCCTTATGACCTGGGC

>BC1__isolate4_Paenibacillus_sp._TPD32

TTGGGCGTAAGCGCGCGCAGGCGGTCAATTAAGTTGGGTGTTTAAGCCCGGGGCTCAACCCCGGTTCGCATCCAAAACTGGTTGACTTGAGTGTAGGAGAGGAAAGTGGAATTCCACGTGTAGCGGTGAAATGCGTAGAGATGTGGAGGAACACCAGTGGCGAAGGCGACTTTCTGGCCTATAACTGACGCTGAGGCGCGAAAGCGTGGGGAGCAAACAGGATTAGATACCCTGGTAGTCCACGCCGTAAACGATGCATACTAGGTGTTGGGGATTCGATTCCTCGGTGCCGAAGTTAACACAGTAAGTATGCCGCCTGGGGAGTACGCTCGCAAGAGTGAAACTCAAAGGAATTGACGGGGACCCGCACAAGCAGTGGAGTATGTGGTTTAATTCGAAGCAACGCGAAGAACCTTACCAGGTCTTGACATCCGGGTGTAAGCACTAGAGATAGTGCCCCTCTTCGGANCACCNNAGACAGGTGGTGCATGGTTGTCGTCAGCTCGTGTCGTGAGATGTTGGGTTAAGTCCCGCAACGAGCGCAACCCTTGATCTTAGTTGCCAGCA

>FACS__isolate5_Pseudomonas_sp

GCGTAAGCGCGCGTAGGTGGCTTGATAAGTTGGATGTGAAATCCCCGGGCTCNACCTGGGAACTGCATCCAAAACTGTCTGGCTAGAGTGCGGTAGAGGGTAGTGGAATTTCCAGTGTAGCGGTGAAATGCGTAGATATTGGAAGGAACACCAGTGGCGAAGGCGACTACCTGGACTGACACTGACACTGAGGTGCGAAAGCGTGGGGAGCAAACAGGATTAGATACCCTGGTAGTCCACGCCGTAAACGATGTCAACTAGCCGTTGGGATCCTTGACATCTTAGTGGCGCAGCTAACGCATTAAGTTGACCGCCTGGGGAGTACGGCCGCCAGGTTA

>FACS__isolate6_Paenibacillus_pocheonensis

GATTATTGGGCGTAAGCGCGCGCAGGCGGTTAATTAAGTTGGGTGTTTAAGCCCGGGGCTCAACCCCGGTTCGCATCCAAAACTGGTTGACTTGAGTGTAGGAGAGGAAAGTGGAATTCCACGTGTAGCGGTGAAATGCGTAGAGATGTGGAGGAACACCAGTGGCGAAGGCGACTTTCTGGCCTATAACTGACGCTGAGGCGCGAAAGCGTGGGGAGCAAACAGGATTAGATACCCTGGTAGTCCACGCCGTAAACGATGCATACTAGGTGTTGGGGATTCGATTCCTCGGTGCCGAAGTTAACACAGTAAGTATGCCGCCTGGGGAGTACGCTCGCAAGAGTGAAACTCAAAGGAATTGACGGGGACCCGCACAAGCAGTGGAGTATGTGGTTTAATTCGAAGCAACGCGAAAAACCTTACCAGGTCTTGACATCCCTCTGACCGGTCTGGAGACAGGCCTTCCCTTCGGGGCAGAGGAGACAGGTGGTGC

>FACS__isolate7_Paenibacillus_qinlingensis

CGCGCGCAGGCGGTCATTTAAGTCTGGTGTTTAATCCTGGGGCTCAACCCCAGTTCGCATCGGAAACTGGATGACTTGAGTGTAAGAGAGGAAAGTGGAATTCCACGTGTAGCGGTGAAATGCGTAGAGATGTGGAGGAACACCAGTGGCGAAGGCGACTTTCTGGCTTATAACTGACGCTGAGGCGCGAAAGCGTGGGGAGCAAACAGGATTAGATACCCTGGTAGTCCACGCCGTAAACGATGCATACTAGGTGTCGGGGATTCGATTTCTCGGTGCCGAAGTTAACACAGTAAGTATGCCGCCTGGGGAGTACGCTCGCAAGAGTGAAACTCAAAGGAATTGACGGGGACCCGCACAAGCAGTGGAGTATGTGGTTTAATTCAAAGCAACGCGAAAAACCTTACCAGGTCTTGACATCCCGATGTAACGCCTAGAGATAGGTGCCCTCTTCGGAGCATTGGAGACAGGTGGTGCATGGTTGTCGTCAGCTCGTGTCGTGAGATGTTGGGTTAAGTCCCG
